# A specific miRNA signature promotes radioresistance of human cervical cancer cells

**DOI:** 10.1186/1475-2867-13-118

**Published:** 2013-11-27

**Authors:** Bin Zhang, Jun Chen, Zhenghua Ren, Yongbin Chen, Jinhui Li, Xia Miao, Yang Song, Tao Zhao, Yurong Li, Yongquan Shi, Dongqing Ren, Junye Liu

**Affiliations:** 1 Department of Radiation Medicine, Fourth Military Medical University, 169 Changle Western Road, Xi’an, Shaanxi 710032, China; 2 Department of Osteology, Tangdu Hospital, Fourth Military Medical University, Xi’an, Shaanxi 710038, China; 3 Department of Oncology, Tangdu Hospital, Fourth Military Medical University, Xi’an, Shaanxi 710038, China; 4 State Key Laboratory of Cancer Biology, Xijing Hospital of Digestive Diseases, Fourth Military Medical University, Xi’an, Shaanxi 710032, China; 5 School of Aerospace Medicine, The Fourth Military Medical University, Xi’an, Shaanxi 710032, China

**Keywords:** Cervical cancer, Radioresistance, miR-630, miR-1246, miR-1290, miR-3138

## Abstract

**Background:**

The mechanisms responsible for cervical cancer radioresistance are still largely unexplored. The present study aimed to identify miRNAs associated with radioresistance of cervical cancer cells.

**Methods:**

The radioresistant cervical cancer cell variants were established by repeated selection with irradiation. The miRNA profiles of radioresistant cells and their corresponding controls were analyzed and compared using microarray. Differentially expressed miRNAs were confirmed by quantitative real-time PCR. Cervical cancer cells were transfected with miRNA-specific mimics or inhibitors. Radiosensitivity of cervical cancer cells were determined using colony-forming assay.

**Results:**

Among the differentially expressed miRNAs, 20 miRNAs showed the similar pattern of alteration (14 miRNAs were overexpressed whilst 6 were suppressed) in all three radioresistant cervical cancer cell variants compared to their controls. A miRNA signature consisting of 4 miRNAs (miR-630, miR-1246, miR-1290 and miR-3138) exhibited more than 5 folds of increase in radioresistant cells. Subsequent analysis revealed that these four miRNAs could be up-regulated in cervical cancer cells by radiation treatment in both time-dependent and dose-dependent manners. Ectopic expression of each of these 4 miRNAs can dramatically increase the survival fraction of irradiated cervical cancer cells. Moreover, inhibition of miR-630, one miRNA of the specific signature, could reverse radioresistance of cervical cancer cells.

**Conclusions:**

The present study indicated that miRNA is involved in radioresistance of human cervical cancer cells and that a specific miRNA signature consisting of miR-630, miR-1246, miR-1290 and miR-3138 could promote radioresistance of cervical cancer cells.

## Background

Cervical cancer is the second largest cause of cancer mortality in women worldwide with more than 270 000 deaths per year [1]. Radiotherapy has a significant role in definitive and adjuvant therapy for cervical cancer. Investigations showed that radiotherapy is used to treat more than 60% of cervical cancer cases [2]. Unfortunately, studies also indicated that the overall incidence of local recurrence is 13% following definitive radiotherapy [3], which suggesting that recurrence after radiotherapy remains a problem in the treatment of cervical cancer. The major obstacle to the treatment success of radiotherapy is radioresistance. Moreover, salvaging previously radioresistant tumors using either radiotherapy or surgery with concern for normal tissue complications is difficult. Therefore, it has significance to reveal the mechanisms underlying radioresistance in cervical cancer.

Some progress has been achieved in the past decades. Increased DNA repair of cancer cells [4] and hypoxia in tumor microenvironment [5,6] have been proposed to be the major reasons for radioresistance. In addition, EGFR [7,8], Cox-2 [9,10], AKT [11], and Her-2 [12] were also suggested playing some roles in radioresistance in cervical cancer in different ways. However, mechanisms responsible for cervical cancer radioresistance are still largely unexplored.

MicroRNAs (miRNAs) are noncoding RNAs of approximate 22 nt in length that function as post-transcriptional regulators. By base-pairing with the complementary sites in the 3′-untranslated region (3′UTR) of the mRNA, miRNAs control mRNA stability and translation efficiency [13-15]. Considering that miRNAs are predicted to regulate translation of a lot of human mRNAs [16], it is no surprise that miRNAs have emerged as important regulators in developmental, physiological and pathological settings including cell growth, differentiation, apoptosis, metabolism and tumorigenesis [17]. More recently, several miRNAs have been demonstrated to be involved in tumor radioresistance. MiR-210 [18], miR-17-92 [19], miR-31 [20], miR-221 and miR-222 [21] have been documented to be dysregulated in radioresistant cancer cells and to promote cancer radioresistance. However, little is known concerning the role of miRNAs in cervical cancer radioresistance.

Driven by these observations, we decided to investigate whether miRNAs play a role in the radioresistance of cervical cancer. We started the present study from establishment of radioresistant cervical cancer cell variants, Hela-R11 and Siha-R15, by repeated selection of Hela and Siha cells with low-dosage of radiation. In the previous study, we have demonstrated that N-Myc downstream-regulated gene 2 (NDRG2) could promote radioresistance of cervical cancer Hela cells [22]. The radioresistant cells Hela-NDRG2 and their control Hela-C cells were also used in this study, which were previously generated by transfection with constructs expressing NDRG2 and control vector respectively in Hela cells [22]. The miRNA profiles of Hela-R11/Hela, Siha-R15/Siha and Hela-NDRG2/Hela-C cells were analyzed with miRNA microarray. A specific miRNA signature was revealed associated with radioresistance of human cervical cancer cells.

## Results

### Establishment of radioresistant cervical cancer cell variants

**Figure 1:**
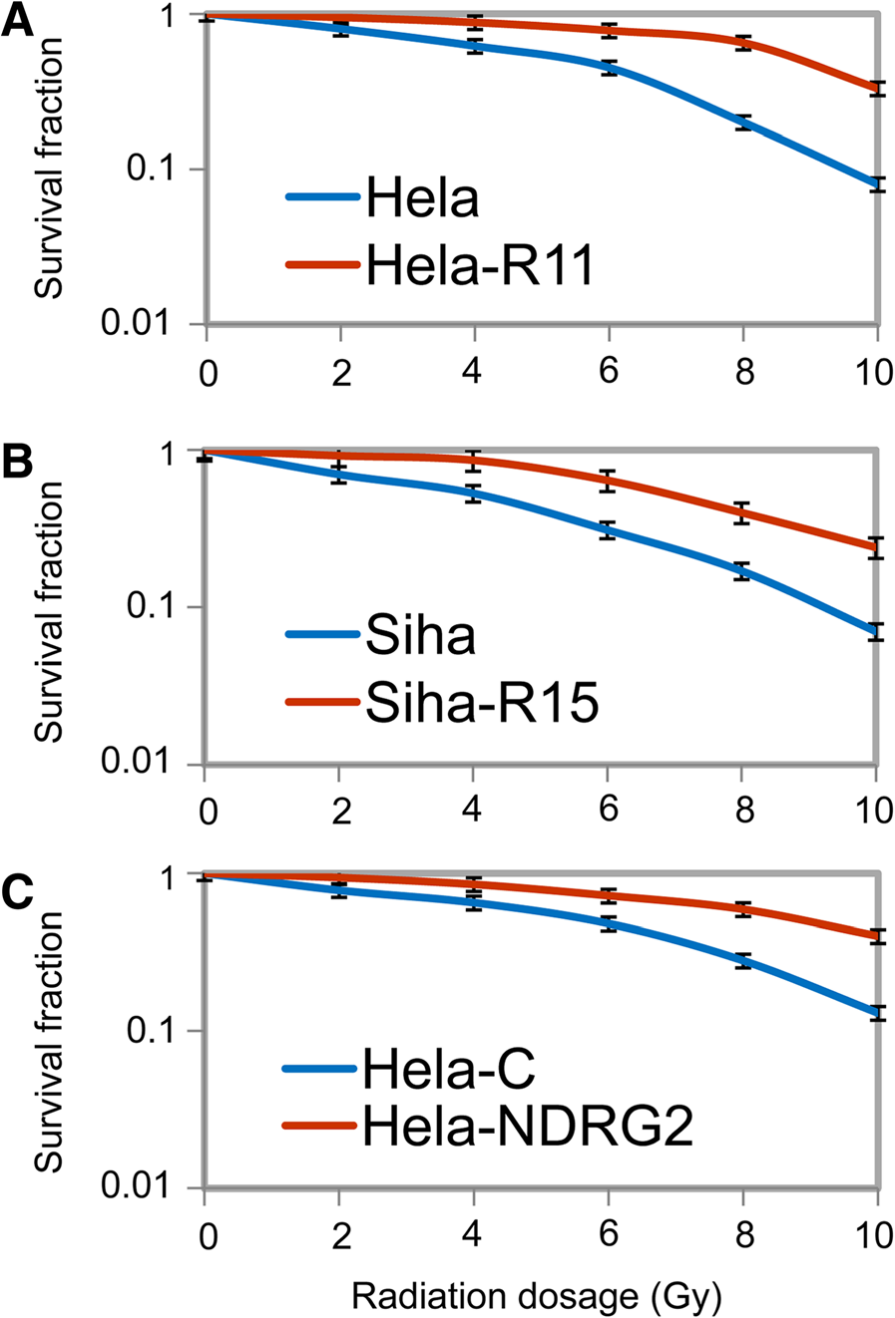
**Three radioresistant cervical cancer cell variants were established.** The radioresistant variants Hela-R11 **(A)** and Siha-R15 **(B)** were established by exposing Hela and Siha cells to 11 and 15 cycles of sequential radiation, respectively. Another radioresistant cervical cancer cells Hela-NDRG2 **(C)** and their control Hela-C cells were generated in our previous study by transfection with constructs expressing NDRG2 and control vector respectively in Hela cells. The cervical cancer cells were exposed to irradiation at indicated dosage and survival fractions were determined by colony-forming assay as described in “Materials and methods”. Data was expressed as mean ± SD of triplicates in one experiment. Shown was representative of at least 3 experiments.

Prior to the analysis of miRNA expression, we first established three couples of human cervical cancer cell lines. One of each couple is radioresistant while another is radiosensitive. The radioresistant Hela-R11 and Siha-R15 cells were derived from their radiosensitive parent cells Hela and Siha by repeated selection with radiation, respectively. Briefly, at the very beginning, the Hela and Siha cells were exposed to 2 Gy of irradiation, which leads to apoptosis of the majority of cells. The rest viable cells were subcultured and expanded in the next 3–5 days. The radiation treatment was repeated when cells reach 60-90% confluency. The apoptosis barely appeared in Hela-R11 and Siha-R15 cells after 11 and 15 cycles of screening, respectively. This result suggested that these two sublines achieved radioresistance. Moreover, we have demonstrated in our previous study that the Hela-NDRG2 cells were radioresistant when compared to Hela-C cells [22]. This couple of cells was generated by transfection with constructs expressing NDRG2 and control vector respectively in Hela cells. In the next step, clonogenic assay was performed to examine the cell survival fractions to further demonstrate the significant differences between radioresistant and control cervical cancer cells. It was shown that the cell survival fractions of Hela-R11, Siha-R15 and Hela-NDRG2 cells were strikingly increased when compared to Hela, Siha and Hela-C cells as their controls respectively (Figure 1). These results indicated that Hela-R11, Siha-R15 and Hela-NDRG2 cell lines were successfully established as the radioresistant cervical cancer cell variants.

Each of these three couple of cells have same origin but with distinctly different radiosensitivities, providing us models to investigate the molecular determinants of responses to radiation in cervical cancer cells, and limit the number of confounding factors, such as inherent genetic variation, that may arise when using cell lines of different origin.

### miRNA profile associated with radioresistance of human cervical cancer cells

**Figure 2:**
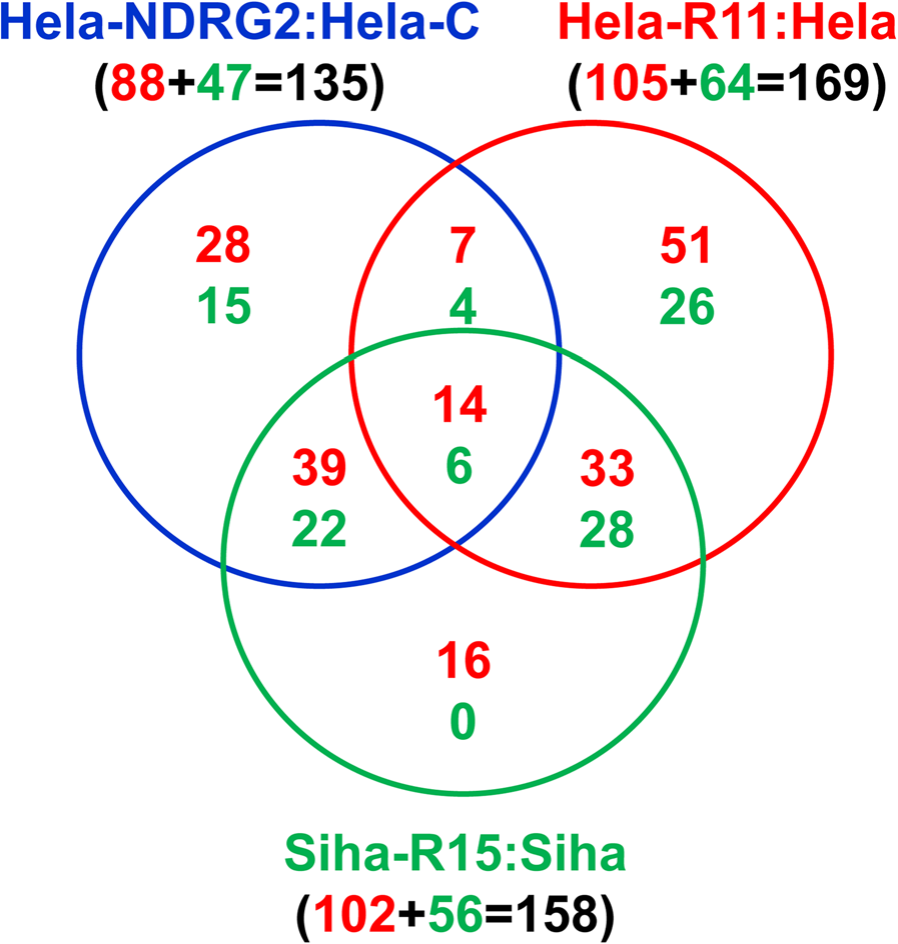
**Human radioresistant cervical cancer cells displayed differential miRNA profiles.** The miRNA profiles of cervical cancer cells were analyzed with miRNA microarray. The profiles of radioresistant cells (Hela-NDRG2, Hela-R11 and Siha-R15) were compared to their corresponding controls (Hela-C, Hela and Siha). Schematic map showed the number of miRNAs with more than 2 folds changes of expression in radioresistant cells (red number denotes up-regulated miRNAs while green number denotes down-regulated miRNAs). The differential miRNA profiles shared by those radioresistant cells were indicated.

**Figure 3:**
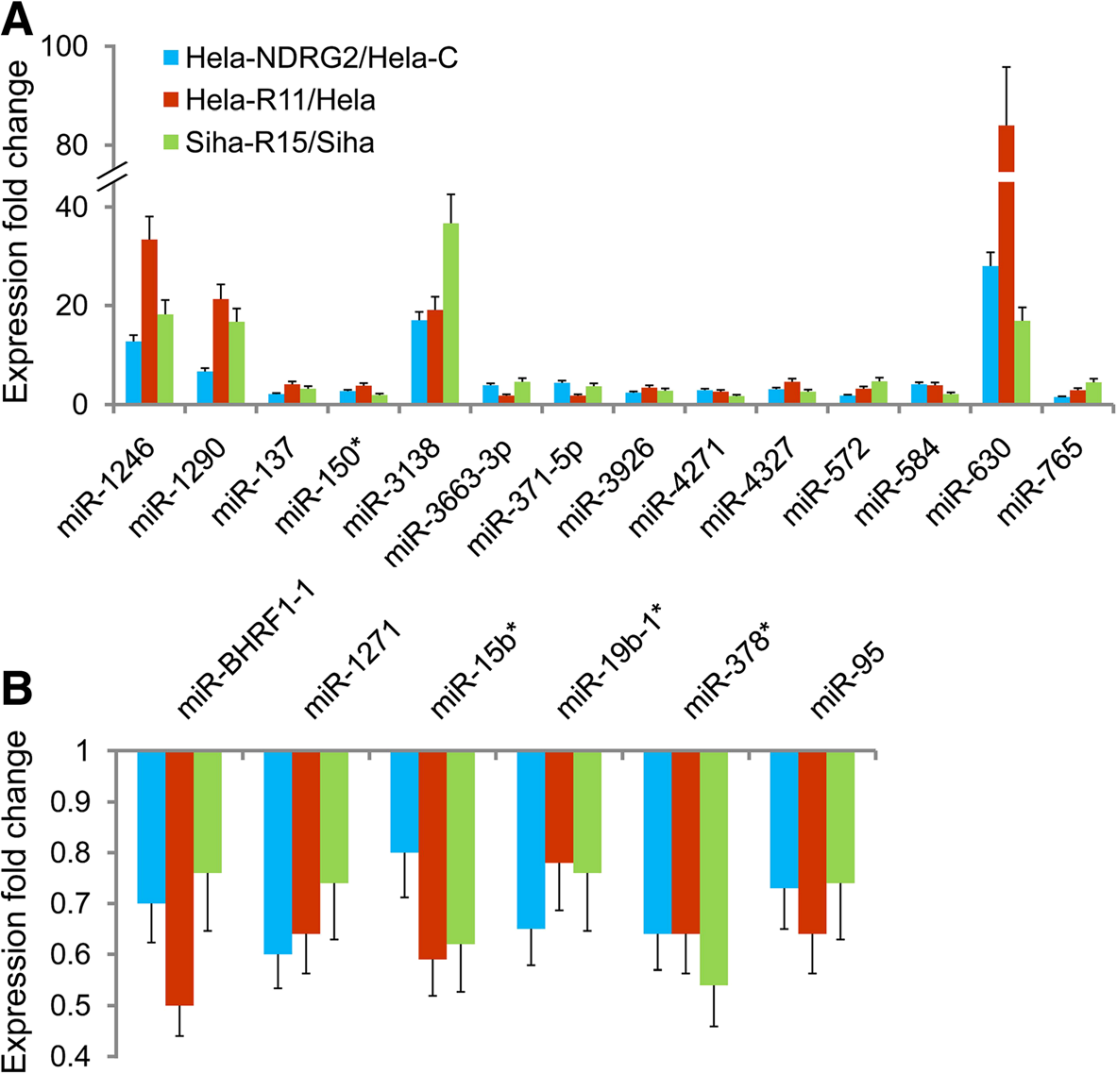
**Twenty miRNAs were differentially expressed in radioresistant cervical cancer cell variants.** Quantitative real-time PCR was performed to determine the expression levels of 14 up-regulated **(A)** and 6 down-regulated **(B)** miRNAs in cervical cancer cells. Data was expressed as fold change of radioresistant variants comparing to their corresponding controls. Shown was representative of 3 independent experiments.

To investigate the radio-specific changes in the microRNAome of radioresistant cervical cancer cells, we assessed the global miRNA expression profile of above three couple of cells by microarray technology. The miRNA expression profile showed that the differences of miRNA expression between radioresistant and radiosensitive human cervical cancer cells were extremely intricate. There were 88, 105 and 102 kinds of miRNAs overexpressed whilst 47, 64 and 56 were suppressed in Hela-NDRG2, Hela-R11 and Siha-R15 cells when compared to Hela-C, Hela and Siha cells as their control respectively. However, it should be noted that there were only 20 miRNAs, of which 14 were up-regulated and others were down-regulated, having the similar alteration in all three radioresistant cervical cancer cell variants compared to their control (Figure 2). Moreover, quantitative real-time PCR (qRT-PCR) assays have been performed in above three couple of cells to determine the expression change of these 20 miRNAs. The results of qRT-PCR were exactly in consistent with microarray that 14 miRNAs were overexpressed whilst 6 were suppressed in all three radioresistant cervical cancer cell variants compared to their control (Figure 3). These data suggested that miRNA profile was associated with radioresistance of human cervical cancer cells.

### Specific miRNA signature is increased upon exposure to radiation in human cervical cancer cells

**Figure 4:**
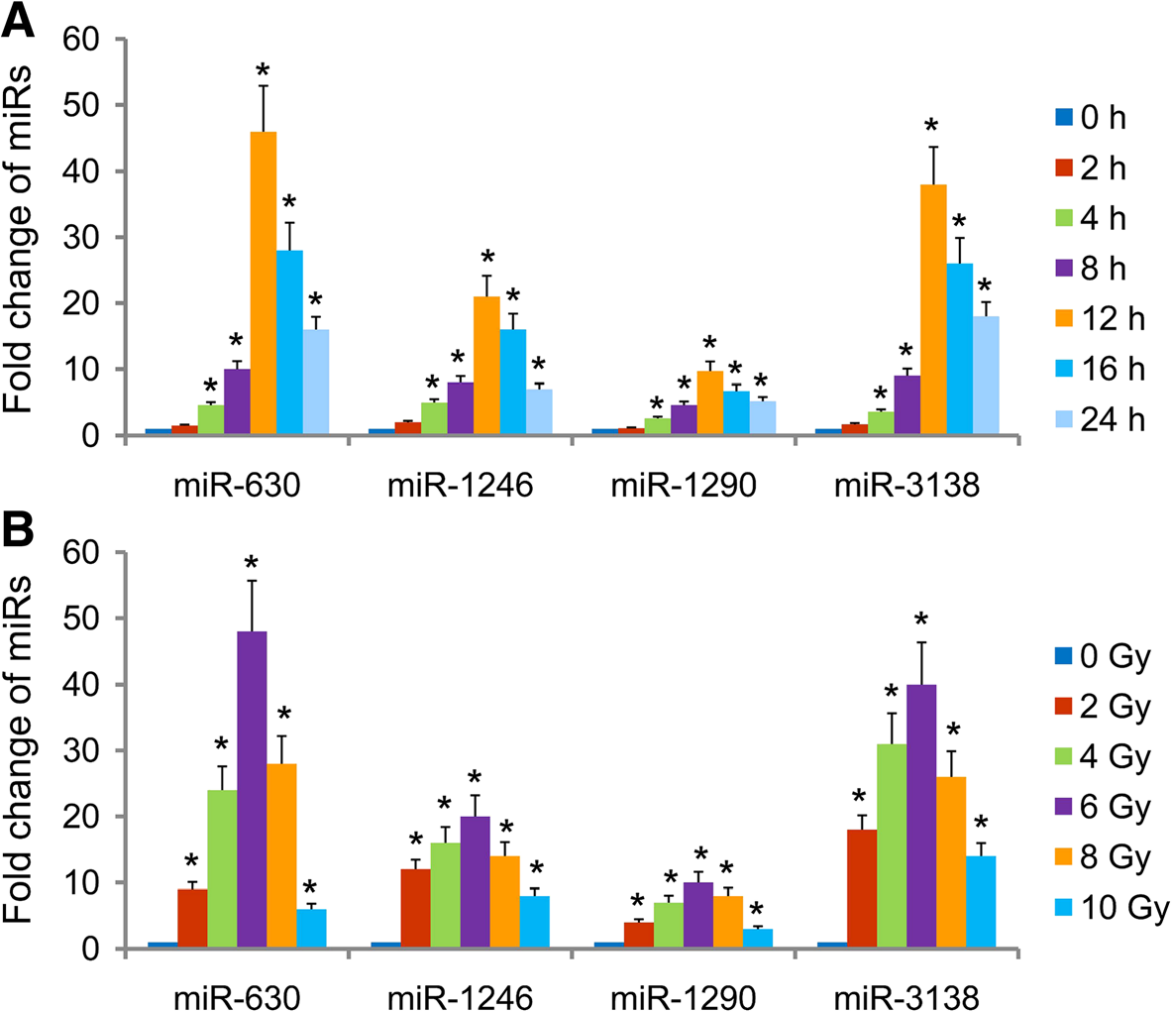
**Radiation induced expression of a miRNA signature in cervical cancer cells in time-dependent and dose-dependent manners.** Hela cells were exposed to irradiation at 6 Gy for desired time **(A)** or at indicated dosage for 12 h **(B)**. The expression levels of miRNAs were evaluated using quantitative real-time PCR. Fold changes of irradiated cells comparing to non-irradiated controls were calculated. Data was expressed as mean ± SD of triplicates in one experiment. Shown was representative of 3 independent experiments. *p < 0.05 vs non-irradiated controls.

Of note, qRT-PCR illustrated that the expression of 4 miRNAs were up-regulated over 5 folds, including miR-630, miR-1246, miR-1290 and miR-3138. Nevertheless, there were no such significant differences in expressions of other miRNAs (Figure 3). Hence, we selected this specific miRNA signature for further investigation. To find out whether radiation could regulate the expression of this specific miRNA signature, we first analyzed the expression of miR-630, miR-1246, miR-1290 and miR-3138 by qRT-PCR in human cervical cancer Hela cells after radiation at a dose of 6 Gy. It was shown that the expression of all these 4 miRNAs was increased upon exposure to radiation in a time dependent manner and sustained at least for 24 h. While the specific miRNA signature expression hasn’t significantly influenced by 2 h of radiation treatment, it was apparently elevated 4 h after radiation, steadily increased to its peak at 12 h and then gradually decreased (Figure 4A). In another set of experiment, Hela cells were irradiated at different dosage and the specific miRNA signature expression was followed 12 h after radiation. It was clear that the expression of these 4 miRNAs could be also up-regulated by radiation in a dose dependent manner. Moreover, 6 Gy of radiation showed the most profound effects on up-regulation of the specific miRNA signature expression, even though all those dosages of radiation had stimulating effects (Figure 4B). The extremely similar results were observed in human cervical cancer Siha cells (data not shown). The above results revealed that this specific miRNA signature is increased upon exposure to radiation in human cervical cancer cells in both time dependent and dose dependent manners, suggesting a potential role of this miRNA signature in radioresistance of cervical cancer cells.

### Specific miRNA signature promotes radioresistance of human cervical cancer cells

**Figure 5:**
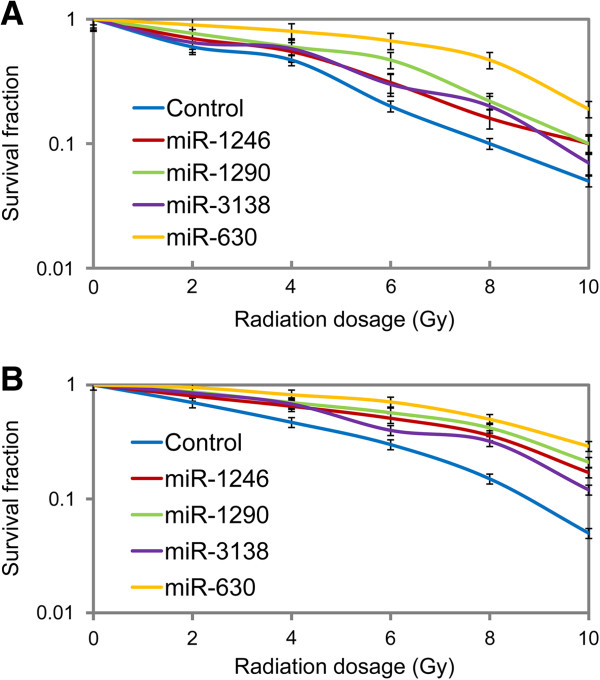
**Specific miRNA signature promoted radioresistance of human cervical cancer cells.** Hela **(A)** or Siha **(B)** cells were transfected with specific miRNA mimics and exposed to irradiation at indicated dosage. The survival fractions were determined by colony-forming assay as described in “Materials and methods”. Data was expressed as mean ± SD of triplicates in one experiment. Shown was representative of 3 independent experiments.

**Figure 6:**
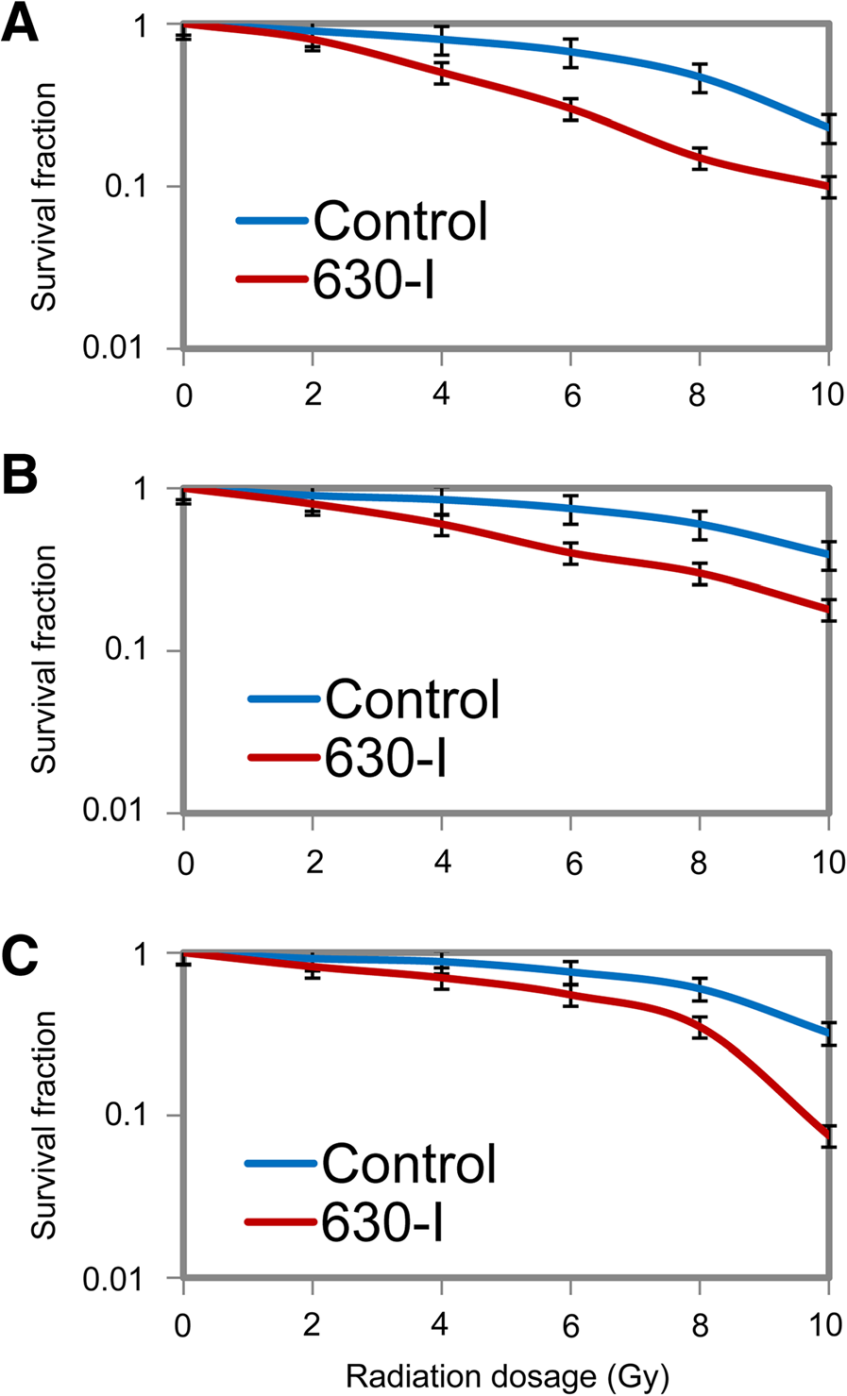
**Inhibition of miR-630 increased radiosensitivity of cervical cancer cells.** The radioresistant cervical cancer cell variants, including Hela-NDRG2 **(A)**, Hela-R11 **(B)** and Siha-R15 **(C)**, were transfected with miR-630 specific inhibitors (630-I) and exposed to irradiation. The survival fractions were determined by colony-forming assay as described in “Materials and methods”. Data was expressed as mean ± SD of triplicates in one experiment. Shown was representative of 3 independent experiments.

To investigate whether the specific miRNA signature is involved in the development of radioresistance of cervical cancer cells, we used Hela cells transfected with the mimics specific for the 4 miRNAs, which respectively express relatively higher miR-630, miR-1246, miR-1290 and miR-3138 than negative control cells (data not shown). After exposure to 0, 2, 4, 6, 8, 10 Gy of irradiation respectively, cell survival fractions were examined using a clonogenic assay to assess the effects of the specific miRNA signature on radiosensitivity. It was shown that overexpression of each of these 4 miRNAs by transfection with their mimic can dramatically increased the survival fraction of irradiated Hela cells (Figure 5A). The results obtained from Siha cells (Figure 5B), which subject to the same treatment, were in accordance with the above results. We noted that the radiosensitivities of Hela and Siha cells transfected with miR-630-mimics were much more significantly attenuated when compared to cells transfected with other miRNAs in the specific miRNA signature (Figure 5A,B). Therefore, the miR-630 was selected for the further experiments as the symbol of the specific miRNA signature. The expression of miR-630 in Hela-NDRG2, Hela-R11 and Siha-R15 cells was suppressed by transfection with miR-630-inhibitors (data not shown). As indicated by the results of clonogenic assay, significantly lower survival fractions were noted in cervical cancer cells with suppressed miR-630 when compared to their controls (Figure 6A,B,C). These suggested that inhibition of miR-630, delegate of the specific miRNA signature, could reverse radioresistance of cervical cancer cells. Taken together, it was indicated that this specific miRNA signature could promotes radioresistance of human cervical cancer cells.

## Discussion

Impaired responsiveness of tumors to radiotherapy is a major clinical problem in cervical cancer. Although extensive studies have been carried out to reveal the mechanisms underlying radioresistance, evidences so far suggest that mechanisms responsible for cervical cancer radioresistance are still not clear and likely to be intricate. Our present data indicated that a specific miRNA signature including miR-630, miR-1246, miR-1290 and miR-3138 could promote radioresistance of human cervical cancer cells. This is the first study suggesting the involvement of miRNAs in the cervical cancer radioresistance, to our knowledge.

miRNAs have been demonstrated to be regulators of many functional genes. It has been shown miR-218 could inhibit cancer cell migration and invasion by targeting focal adhesion pathway [23], impair tumor growth and increase chemo-sensitivity to cisplatin through regulating the AKT-mTOR signaling pathway [24] and suppress progression through downregulation of survivin and the SLIT2-ROBO1 pathway [25]. miRNA microarray analysis has been performed to compare the differences of miRNA profiles between cervical cancer tissues and normal cervical tissues [26,27] or adjacent normal cervical tissues [28], suggesting important roles of specific miRNAs in cervical tumorigenesis. However, miRNA function depends on the particular tissue type in which they are found and the cellular environment in which they are expressed [29]. In the present study, miRNA microarray was employed to compare the miRNA profiles of 3 couples of cervical cancer cell variants derived from different origin, of which one is radioresistant and another is the corresponding control. This strategy helps us to omit the prior influence of tissue type and cellular environment on miRNA expression pattern and clarify the mechanisms of human cervical cancer radioresistance in a way. Although altered expression of vast miRNAs in radioresistant cervical cancer cell models was indicated, only a limited miRNA profile, that contains 14 miRNAs which were up-regulated and 6 others suppressed, has been shared by all three radioresistant cell variants. Hela-NDRG2 and Hela-R11, deriving from the same cell line, have 31 miRNAs overlapped. This may reflect different influence of generation methods on miRNA expressing profiles. Hela-NDRG2 was generated by overexpressing NDRG2 in Hela cells, while Hela-R11 was established by repeated exposure to radiation for 11 times. On the other hand, the overlapped miRNAs have the bigger possibility to be the drivers while other miRNAs may be passengers in responding to radiation. These strongly indicate that some miRNAs are specific to and involved in radioresistance of cervical cancer cells.

Several miRNAs have been demonstrated to be involved in radioresistance as suppressors. miR-210 [18], miR-17-92 [19], miR-31 [20], miR-221 and miR-222 [21] have been documented to be down-regulated in radioresistant cancer cells, to regulate the expression of AIFM3, MNT and PTEN respectively, and to promote cancer radioresistance. In the present study, 4 miRNAs (miR-630, miR-1246, miR-1290 and miR-3138) showing more than 5 folds of expression changes were selected for further analysis. This specific miRNA signature is increased upon exposure to radiation in human cervical cancer cells in a time dependent and a dose dependent manners. Most recently, Chaudhry et al. [30] reported the identification of radiation-induced miRNA transcriptome by next-generation massively parallel sequencing in human lymphoblast cell TK6. It was shown that more than 30 miRNAs are modulated by irradiation. Interestingly, 12 miRNAs exhibited two peaks of induction while 15 miRNAs were induced only at one time point [30]. In another study, the same group analyzed radiation-induced miRNA modulation in glioblastoma cells M059J and M059K, two cell lines from the same origin. M059J is deficient in DNA-dependent protein kinase whereas M059K has normal kinase activity. The miR-17-3p, miR-17-5p, miR-19a, miR-19b, miR-142-3p, and miR-142-5p were upregulated in both M059K and M059J cells [31]. However, the miR-15a, miR-16, miR-143, miR-155, and miR-21 were upregulated in M059K, and the modulation of these miRNAs fluctuated in M059J cells in a time-dependent manner [31]. The dynamic miRNA profiles in M059 cells are different from that in TK6 cells. The radiation-induced miRNAs (miR-630, miR-1246, miR-1290 and miR-3138) in cervical cancer cells are not included in the miRNA profiles of M059 and TK6, supporting the speculation that the modulation of miRNA is dependent on cell type, radiation dose and dose rate [30].

The present study also demonstrated that overexpression of the specific miRNA signature (miR-630, miR-1246, miR-1290 and miR-3138) by transfection with its mimics respectively could enhance the radioresistance in cervical cancer cells, and that suppression of miR-630, delegate of the specific miRNA signature, attenuates the radioresistance in cervical cancer cells. These results suggest that overexpression of this specific miRNA signature may be important for surviving the cytotoxic effects of radiation and promotes radioresistance of human cervical cancer cells. It has been reported that miR-630 regulates cisplatin-induced cancer cell death [32] and acts as a regulator downstream of phospho-ΔNp63α in autophagy [33]. miR-1246 can be up-regulated in human epidermoid carcinoma cells A431 in response to photodynamic therapy [34], serve as a novel diagnostic and prognostic biomarker for oesophageal squamous cell carcinoma [35] and multiple myeloma [36]. Up-regulation of microRNA-1290 impairs cytokinesis and affects the reprogramming of colon cancer cells [37]. The present study reveals positive roles of miR-630, miR-1246, miR-1290 and miR-3138 in radioresistance of cervical cancer cells, supporting regulatory roles of miRNAs in radioresistance. However, it should be noted that the targets of these miRNAs and the relative pathways need further exploration.

## Conclusions

In conclusion, our findings suggest that miRNAs may play a role in radioresistance of cervical cancer and a specific miRNA signature promotes radioresistance of human cervical cancer cells. These data can help us to better understand the molecular mechanisms underlying resistance to radiation and the role of miRNAs in development of cancer radioresistance. Further studies are encouraged to reveal the targets and mechanisms underlying the specific miRNA signature in radioresistance and to evaluate the clinical relevance and significance of this specific miRNA signature in cervical cancer.

## Materials and methods

### Cell culture and transfection

The human cervical cancer cell lines Hela and Siha were obtained from the American Type Culture Collection (Manassas, VA). Hela variants Hela-NDRG2 and Hela-C were previously established by stable transfection with constructs expressing NDRG2 and control vector respectively in Hela cells [22]. All the cells were maintained as a monolayer in Dulbecco’s modified Eagle’s medium (Invitrogen, Carlsbad, CA) supplemented with 10% fetal bovine serum (Sijiqing Biological Engineering Materials Co., Hangzhou, China) at 37°C in the presence of 5% CO_2_-balanced air.

The mimics and inhibitors specific for miR-630, miR-1246, miR-1290 and miR-3138 were obtained from Ambion (Grand Island, NY). Cervical cancer cells were transfected with the miRNA mimics or inhibitors at a final concentration of 100 μM (mimics) or 200 μM (inhibitor) using LipofectamineTM 2000 (Invitrogen, Carlsbad, CA) according to the manufacturer’s instruction.

### Irradiation

Irradiation was performed using a ^60^Co γ-ray therapeutic machine, RCR-120 (Toshiba, Tokyo, Japan), at a dose rate of 1.6 Gy/min.

### Colony-forming assay

The radiosensitivity of cervical cells was determined using colony-forming assay as previously described [22]. In brief, cancer cells at 60-80% confluency were trypsinized into single cell suspension and exposed to desired dosage of irradiation. Following exposure, cells (200–2000) were plated in 60 mm dishes and incubated at 37°C, 5% CO_2_ for colony formation. After 10–14 days of growth, the colonies were fixed with 10% (v/v) methanol for 15 min and stained with 5% (g/v) Giemsa solution (Sigma) for 20 min. Colonies that consisted of more than 50 cells were scored. Colony plating efficiency was calculated to be the number of viable plated cells, and expressed as a percentage of inoculated cells. In each group, survival fraction of cells was calculated as plating efficiency of the irradiated cells divided by the plating efficiency of the untreated control. Survival curves were plotted as the log of survival fraction versus radiation dosage.

### miRNA microarray analysis

The miRNA profiles of three couple of cells (Hela-R11/Hela, Siha-R15/Siha and Hela-NDRG2/Hela-C) were analyzed using miRNA microarray. Total RNA from cervical cancer cells was isolated with Trizol reagent (Invitrogen, Carlsbad, CA) and miRNA fraction was further purified using a mirVanaTM miRNA isolation kit (Ambion, Austin, TX). The isolated miRNAs from the 2 cell lines of each couple were then labeled with Hy3 using the miRCURYTM Array Labelling kit (Exiqon, Vedbaek Denmark) and hybridized respectively on a miRCURYTM LNA microRNA Array (v 8.0, Exiqon) as described [38]. Microarray images were acquired using a Genepix 4000B scanner (Axon Instruments, Union City, CA) and processed and analyzed with Genepix Pro 6.0 software (Axon Instruments) and Excel.

### Real-time PCR

The expression levels of miRNAs in cervical cancer cells were determined using real-time PCR. Total RNA of cervical cancer cells was extracted using Trizol reagent (Invitrogen, Carlsbad, CA) and reverse transcription was performed according to the manufacturer’s instructions (D350A, TaKaRa Biotechnology, Dalian, Liaoning, China). Quantitative real-time PCR (qRT-PCR) was performed to determine the expression levels of each miRNA using the exact sequences (U to T) of these miRNAs as the forward primer and the unique q-PCR primer from the cDNA Synthesis Kit. U6 was used as an internal control, and each plate contains one cDNA sample for each primer as a correct sample.

### Statistical analysis

Data are expressed as mean ± SD. Statistical analyses were performed with the SPSS software (version 10.0; SPSS, Chicago, IL) by using one-way ANOVA followed by the t-test for independent groups. A p level of < 0.05 was considered statistically significant.

## Abbreviations

miRNA: microRNA; NDRG2: N-Myc downstream-regulated gene 2; qRT-PCR: Quantitative real-time PCR.

## Competing interests

The authors declare that they have no competing interests.

## Authors’ contribution

BZ carried out the miRNA microarray analysis and participated in cell transfection. JC carried out colony-forming assay and cell culture. ZR and JL performed real-time PCR and took part in colony-forming assay. XM participated in cell culture and transfection. YS participated in real-time PCR and miRNA microarray analysis. TZ performed irradiation. YL performed the statistical analysis. YC conceived the study and participated in miRNA microarray analysis. YS participated in the study design and helped to draft the manuscript. JL and DR conceived the study, carried out its design and drafted the manuscript. All authors read and approved the final manuscript.
